# Manchester and the Wounded

**Published:** 1916-01-01

**Authors:** 


### Manchester and the Wounded.

MANCHESTER, which already has a larger number of wounded soldiers under treatment within its confines than any other city in the United Kingdom, outside of London, bids fair to become an even more important centre for the care of those broken in the war.

The War Office has made a demand for the Withington Poor-Law Infirmary—one of the largest in the country—and the Manchester Guardians have placed the greater part of the premises, together with the nursing staff, etc., at the disposal of the authorities. In order that a total of over 2,000 beds may be offered, all but a few of the oldest and feeblest inmates are to be removed elsewhere.

Accommodation for 4,000 convalescent men has also been provided at Heaton Park, where full use is being made of light treatment and electric massage, for which purposes the library and organ room have been set apart.

The military authorities have now come to the conclusion that it would be better from every point of view to concentrate invalid soldiers in one centre in Lancashire instead of having them scattered throughout the county. It is therefore proposed to utilise Platt Fields Park, which covers nearly 80 acres in one of the Manchester suburbs. The scheme is to erect there from 3,000 to 4,000 huts, and to provide accommodation at first for 2,000 wounded soldiers, to be increased eventually to 10,000. It is intended that the occupants of the beds shall be mainly convalescents, and that the scheme shall be worked in close co-operation with the Manchester Royal Infirmary and its large staff. The large refreshment room in the park will be used as doctors' and officers' quarters.

